# Burnout and intent to leave during COVID‐19: A cross‐sectional study of New Jersey hospital nurses

**DOI:** 10.1111/jonm.13647

**Published:** 2022-05-11

**Authors:** Pamela B. de Cordova, Mary L. Johansen, Irina B. Grafova, Suzanne Crincoli, Joseph Prado, Monika Pogorzelska‐Maziarz

**Affiliations:** ^1^ Rutgers, the State University of New Jersey, Division of Nursing Science – School of Nursing Division of Nursing Science, School of Nursing Newark New Jersey USA; ^2^ New Jersey Collaborating Center for Nursing (NJCCN) Newark New Jersey USA; ^3^ Rutgers, the State University of New Jersey Division of Entry to Baccalaureate Nursing, School of Nursing Newark New Jersey USA; ^4^ Rutgers, the State University of New Jersey Minority Biomedical Research Support Program (MBRS) Newark New Jersey USA; ^5^ Jefferson College of Nursing, Thomas Jefferson University Philadelphia Pennsylvania USA

**Keywords:** burnout, COVID‐19, hospitals, intent to leave

## Abstract

**Aim:**

The aim of this work is to examine staffing, personal protective equipment (PPE) adequacy and physical exhaustion that contributed to burnout and intent to leave among hospital nurses during the first peak of the COVID‐19 pandemic.

**Background:**

Burnout is associated with adverse nurse and patient outcomes. Identifying the magnitude of burnout that occurred during the pandemic can prepare managers for the long‐term mental health effects on nurses.

**Methods:**

A cross‐sectional, electronic survey was administered to examine perceptions of burnout and intent to leave among all New Jersey hospital nurses from October 6 to October 26, 2020.

**Results:**

A total of 3030 nurses responded with 64.3% reporting burnout and 36.5% reporting intent to leave the hospital within a year. There was a significant association between high levels of burnout and intent to leave (*χ* ^2^ = 329.4; *p* = .001). There was no association between staffing and burnout; however, reporting inadequate PPE (OR = 1.77 [95% CI: 1.34–2.34]) and physical exhaustion (OR = 3.89 [95% CI: 3.19–4.76]) remained predictors of burnout among nurses.

**Conclusion:**

Inadequate PPE and physical exhaustion coupled with short staffing contributed to burnout and intent to leave.

**Implications for Nursing Management:**

Managers should continue to utilize evidence‐based mental health interventions and advocate within their nursing professional organizations for relief funds to reduce burnout.

## 1 INTRODUCTION

In March 2020, health care providers in the United States began to grapple with the COVID‐19 pandemic caused by the severe acute respiratory syndrome coronavirus 2 (SARS‐CoV‐2) (Schuchat, 2020). During the early months, nurses cared for their patients while dealing with fear of exposure to COVID‐19 and the uncertainty regarding the availability and allocation of resources including staffing and personal protective equipment (PPE). Among all frontline acute providers, registered nurses (RN) had the most direct, extensive frequent contact with patients, which placed them at disproportionately greater risk of exposure (Cohen & van der Meulen Rodgers, 2020; Lai et al., 2020).

## 2 BACKGROUND

A seminal study examining hospital nurse staffing and burnout defines burnout as an occupational condition with emotional exhaustion, depersonalization, and low job satisfaction (Aiken et al., 2002). In a meta‐analysis of nurse staffing and burnout, a greater nurse‐to‐patient ratio was consistently associated with a higher degree of burnout (Shin et al., 2018). Inadequate staffing from greater workload, extra shifts, and physical exhaustion were found to be predictors of burnout among clinical nurses (Rozo et al., 2017).

During the peaks of waves of the pandemic, the effects of inadequate staffing were exacerbated as hospitals surged with COVID‐19 patients. During this time, nurses reported a higher level of anger, depression, and anxiety with increased workload as they provided care during the pandemic (Shen et al., 2021). In a recent systematic review and meta‐analysis, researchers found that factors that increased nurses' burnout included working in high‐risk environments, working in hospitals with inadequate PPE, increased workload, and lower level of training for caring for COVID‐19 patients (Galanis et al., 2021).

Reducing burnout and intent to leave is important for nurses' mental health and mitigates shortages that may be exacerbated by the pandemic. Nurse burnout is associated with adverse patient outcomes, medical errors, decreased patient satisfaction, and intent to leave the profession in the hospital setting (Brooks Carthon et al., 2021; Hämmig, 2018). Intent to leave is defined as the likelihood that nurses would leave their current hospital position for another nursing position outside the hospital setting and the probability of permanently leaving the organization (Gebregziabher et al., 2020; Phillips, 2020). Nurses who intend to leave their jobs because of burnout report a stressful work environment and inadequate staffing as major factors (Shah et al., 2021). Evidence from burnout and intent to leave during the first wave can help managers to develop better strategies and to prepare the workforce for subsequent waves and future pandemics (Galanis et al., 2021; Shah et al., 2021).

As we approach almost 2 years into this pandemic, there is a need to empirically determine the magnitude of nurse burnout and intent to leave among clinical nurses. However, the extent to which data‐based research has identified factors, such as staffing, PPE adequacy, and physical exhaustion that may have contributed to nurse burnout and intent to leave during the COVID‐19 pandemic is limited. Although evidence is emerging about COVID‐19 burnout, none of the existing evidence link burnout and intent to leave considering these factors. Therefore, the purpose of this study was to examine the association between RN staffing, PPE adequacy, and physical exhaustion on burnout and intent to leave among frontline acute care RNs during the first peak of the COVID‐19 pandemic in New Jersey (NJ).

## 3 METHODS

### 3.1 Sample

In October 2020, we conducted a cross‐sectional survey of 135,253 actively licensed RNs (New Jersey Collaborating Center for Nursing, 2020) during the first peak of COVID‐19, defined as the time period between 13 March 2020 until 1 June 2020 in the state of NJ (New Jersey COVID‐19 Hub, 2021). Prior to any data collection, we obtained approval by the State University of New Jersey Institutional Review Board Pro 20200001775 under minimal risk and exempt category 2 based on Title 45, Part 46.101(b) of the Code of Federal Regulations. We sent recruitment emails to 107,477 actively licensed RNs who provided an e‐mail address to the NJ Board of Nursing and excluded nurses who did not have a mailing address in either NJ, New York, Pennsylvania, Connecticut, and Delaware based on the logic that RNs had to be geographically close to work in a NJ hospital during the first wave. This sampling strategy likely eliminated travel RNs. We targeted RNs who provided direct patient care in an emergency department (ED), observation or an adult, inpatient unit at a NJ acute care hospital during the first peak of COVID‐19. To ensure that nurses worked in hospitals during the peak of the first wave, we excluded those RNs who did not work in acute care, did not work in a NJ hospital and whose primary role during the peak was of an advanced practice nurse.

In 2020, there were 20,179 RNs working in hospitals who provide direct patient care in NJ (New Jersey Collaborating Center for Nursing, 2020). To ensure a representative sample, we compared our survey respondent demographics to the demographics of NJ workforce. Of the 5880 RNs who consented to participate in the survey, 3030 nurses met our inclusion criteria.

### 3.2 Setting

With a population of approximately 9 million people, NJ was one of the states most impacted by COVID‐19 in March 2020. At the peak of first wave on 18 May 2020, there were 3153 hospitalized COVID‐19 patients among 71 hospitals (The New York Times, 2021). On 6 October 2020, there were 209,342 cumulative cases of COVID‐19 with 601 hospitalized patients and 67 patients on ventilators and within three weeks there were 957 hospitalized patients and 80 patients on ventilators on indicative of the beginning the second wave (New Jersey Department of Health, 2021). COVID‐19 vaccinations became available in NJ on 15 December 2020, and there were no official state policies in place to assist the workforce, yet some hospitals offered resiliency trainings.

### 3.3 Survey instrument

We created a self‐administered, electronic survey (available upon request) composed of five main components including the following: (1) Staffing; (2) PPE adequacy; (3) Physical exhaustion; (4) Burnout; (5) Intent to leave. We included baseline demographic characteristics such as identified gender, age, race, ethnicity, and nursing specialty because staffing ratios differ by specialty. To establish content validity, we piloted the survey among eight clinical RNs that worked in a NJ hospital during the first wave. Based on that process, we removed several questions to reduce the length. Based on the pilot, the survey took an average of 12 min to complete.

### 3.4 Measures

#### 3.4.1 Staffing

We measured staffing by asking RNs to self‐report the number of patients they were assigned in a typical shift prior to and during the peak of [the first wave] of the COVID‐19 pandemic. We also asked RNs the safest number of non‐COVID‐19 and COVID‐19 patients they should be assigned in a typical shift. Nurse staffing by self‐report of nurse staffing is validated and more reliable than using administrative data sources in which administrative datasets tend to calculate nurses that do not provide direct patient care (Aiken et al., 2010).

### 3.5 PPE adequacy

We developed four main questions to measure PPE adequacy because of the lack of psychometrically tested measures of PPE adequacy at the time. We asked RNs to identify what PPE they lack on their unit as a select all that apply with nine options including (1) face shields, (2) gloves, (3) goggles/eye protection, (4) gowns, (5) hair protection, (6) N95 respirators, (7) shoe covers, (8) surgical masks, and (9) sufficient supplies. We collapsed those eight categories and recoded the variable into lacking on whether an item was selected or not lacking on whether the item was missing, or “sufficient supplies” was checked. We then asked RNs if they had to ration and reuse with other options (i.e., expired, use donated, and use their own) also as a select all that apply and repeated the same process as above. Using a 5‐point Likert scale, RNs identified how confident that the PPE they used protected them from COVID‐19 transmission and how confident they were the hospitals had adequate PPE for them. We dichotomized these confidence measures into little or no confidence (≤3) and confident as >3.

### 3.6 Physical exhaustion

We created a physical exhaustion scale based on a numeric rating scale from 1 to 10 for pain assessment that clinicians frequently use, with high psychometrics for reliability and validity (Karcioglu et al., 2018). We dichotomized physical exhaustion as ≥7 out of 10 on the numeric scale.

### 3.7 Burnout

We measured burnout by the Dolan single‐item measure that is reliable and validated among primary care staff (Dolan et al., 2015). The single‐item measure has a sensitivity of 83.2%, specificity of 87.4%, positive predictive value of 79.3%, and negative predictive value of 90.0% with the area under the curve of .93 (Dolan et al., 2015). Although Dolan et al. tested this measure among primary care staff, other researchers have validated the instrument among clinical nurses (Edwards et al., 2018). Using a 5‐point Likert scale, RNs were asked to identify their symptoms of burnout by (1) “I enjoy my work. I have no symptoms of burnout”; (2) “Occasionally I am under stress, and I don't always have as much energy as I once did, but I don't feel burned out”; (3) “I am definitely burning out and have one or more symptoms of burnout, such as physical and emotional exhaustion”; (4) “The symptoms of burnout that I'm experiencing won't go away. I think about frustration at work a lot”; (5) “I feel completely burned out and often wonder if I can go on. I am at the point where I may need some changes or may need to seek some sort of help.” Following existing methodology to conduct multivariable logistic regression models, we dichotomized this measure into no symptoms of burnout (≤2) and “burning out/burnt out” as ≥3 (Harris et al., 2018).

### 3.8 Intent to leave

Staff nurse intent to leave as a single item measure has strong reliability (Shang et al., 2013). We measured intent to leave by asking whether RNs intended to leave their current position within the next year (yes/no).

### 3.9 Data collection

We followed the Dillman method for recruitment which recommends personalized, repeated contacts to increase response rates (Dillman et al., 2014). We sent an initial invitation email followed by two reminder emails to noncompleters. To increase recruitment, we offered participants the opportunity to enter a lottery for 70 $100 Amazon electronic gift cards. We obtained online consent from all participants as approved by the IRB. The survey was open for 20 days from 6 October through 26 October 2020.

### 3.10 Analysis

We used *t‐*test and chi‐square tests to examine differences between our RN study sample and the overall RN acute care workforce in NJ by age, gender identity, and race and ethnicity. We used descriptive statistics to identify the mean staffing ratios by specialty (i.e., ED, intensive care unit [ICU], step down unit [SDU], and medical/surgical units) both prior to and at the peak of the pandemic. We used *t‐*tests to test the difference between the mean number of patients assigned and the perceived number of the safest patients that should be assigned to an RN by specialty prior to the peak. We then repeated that analysis to test the difference between number of COVID‐19 patients assigned and the number of safest COVID‐19 patients by specialty. We used chi‐square test to examine associations between the PPE variables, physical exhaustion, burnout, and intent to leave by specialty. We then tested the associations between the PPE variables and intent to leave. We also used chi‐square test to test associations between physical exhaustion and burnout by intent to leave, respectively. We used multivariable logistics regression to measure the associations between burnout and each measure of staffing, PPE adequacy, and physical exhaustion while controlling for hospital specialty. All statistical analyses were performed using STATA version 16.0 (Stata LP, College Station, Texas).

## 4 RESULTS

### 4.1 Nurse demographics

Following the STROBE checklist for cross‐sectional reporting (von Elm et al., 2007), our final sample included 3030 RNs (15% response rate). The comparison of respondents to the general NJ acute care RN workforce reflected a younger demographic (39 years compared with 45 years, *p* < .05) with fewer males, (9.4% compared with 11.7%, *p* < .05), fewer participants that identified as Black (6.1% compared with 8.0%, *p* < .05) and as Asian (11.6% compared with 17.9%, *p* < .05) and a greater proportion that identified as Hispanic as compared with the 2020 NJ RN Workforce (8.4% compared with 6.0%, *p* < .05) (Table 1).

**TABLE 1:** Nurse demographics compared with state workforce

	Survey		State workforce		*p* < .05
*n*	*N* = 3030		*N* = 20,179		
Age, mean (SD), y	38.7	12.3	44.7	13.2	***
**Identified gender**	***n***	**%**	***n***	**%**	
Female	2331	76.9	17,815	88.3	***
Male	283	9.3	2364	11.7	***
Other, transgender, genderqueer	6	0.2	0	0.0	
Prefer not to disclose	410	13.5	0	0.0	
**Identified race**					
American Indian, non‐Hispanic	4	0.1	14	0.1	
Asian	376	12.4	3621	17.9	***
Black or African American	207	6.8	1619	8.0	***
Native Hawaiian/Pacific islander	17	0.6	187	0.9	
White	1746	57.6	10,754	53.3	
Multiple/other	188	6.2	563	2.8	***
Prefer not to disclose	492	16.2	3421	17.0	
**Identified ethnicity**					
Hispanic/Latinx	255	8.4	1201	6.0	***
Non‐Hispanic/Latinx	2202	72.7	16,758	83.0	
Prefer not to disclose	573	18.9	2220	11.0	

### 4.2 Nurse staffing

**FIGURE 1:**
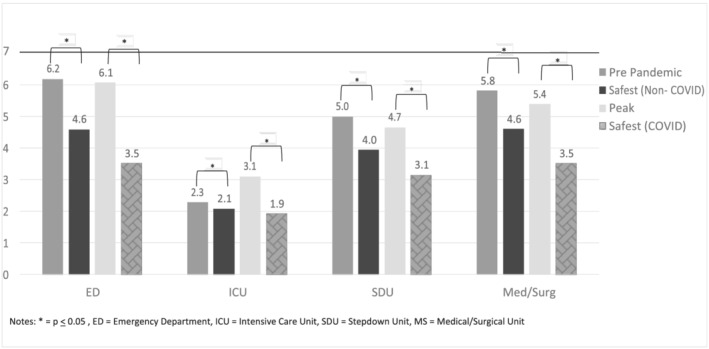
Number of patients assigned during COVID‐19. Notes: *p < .05, ED = emergency department, ICU = intensive care unit, SDU = stepdown unit, MS = medical/surgical unit

Prior to the pandemic, RNs reported that they were assigned, on average, six patients in the ED and medical/surgical units, five patients on the step‐down units, and two patients in the ICU (Figure 1). For all four specialties, the perceptions of what RNs perceived as a safe number of non‐COVID‐19 patients significantly differed from what they were assigned (*p* < .05). Similarly, the perceptions of what RNs perceived as a safe number of COVID‐19 patients that they should be assigned significantly differed from what they were assigned (*p* < .05). The number of patients assigned per RN was similar at the peak as compared with prior to the pandemic except for the ICU in which the staffing ratio in the ICU increased by almost one full patient to a ratio of one RN to three COVID‐19 patients at the peak.

### 4.3 PPE inadequacy, physical exhaustion, burnout, and intent to leave by specialty

Almost 80% (*n* = 2394) reported lacking PPE and nearly 90% (*n* = 2642) reported needing to ration and reuse PPE (Table 2). Almost 75% (*n* = 2228) of the RNs lacked confidence that the hospital had adequate PPE. When examining by specialty, medical/surgical RNs lacked the most confidence in PPE protection and PPE adequacy and reported the need to ration, reuse, or use nonhospital PPE most frequently. The ICU RNs reported the highest level of physical exhaustion as compared with other specialties. The ED RNs reported the highest percentage of burnout (64.3%, *n* = 1908). Among all specialties, 36.5% of the RNs (*n* = 1106) reported their intent to leave within 12 months with medical‐surgical RNs demonstrating the highest percentage of intent to leave.

**TABLE 2:** PPE inadequacy, physical exhaustion, burnout and intent to leave by specialty

	Total		ED		ICU		MS		Other^a^		*χ* ^2^
	*n*	%	*n*	%	*n*	%	*n*	%	*n*	%	
Lacked PPE^b^	2394	79.0	343	77.8	859	82.7	966	84.1	226	71.2	145.1*
Rationed and reused PPE^b^	2642	87.2	384	87.1	927	89.2	1069	90.3	262	87.6	214.0*
Lack of confidence in PPE protection	1974	65.1	286	64.9	675	65.0	828	69.9	185	57.3	66.1*
Lack of confidence in PPE adequacy	2228	73.5	323	73.2	777	74.8	926	78.2	202	64.8	104.8*
Physical exhaustion	2426	80.1	330	74.8	876	84.3	982	82.9	238	71.5	42.9*
Burnout	1908	64.3	294	66.7	659	63.4	769	65.0	186	58.1	26.9*
Intent to leave within 12 months	1106	36.5	167	41.4	378	39.3	477	43.3	84	30.0	31.7*

Abbreviations: ED, emergency department and includes observation units; ICU, intensive care unit and includes step‐down units, MS, medical/surgical unit; PPE, personal protective equipment.

^a^ Other = Maternal health units, recovery room, reassigned units.

^b^ PPE included face shields, gloves, gowns, eye protection, caps, N95s, shoe covers, and surgical masks.

* *p* < .05.

### 4.4 PPE adequacy, physical exhaustion, and burnout by intent to leave

We observed significant differences between lacking PPE (*χ* ^2^(2) = 26.0; *p* = .001), lack of confidence in PPE protection (*χ* ^2^(2) = 61.3; *p* = .001), and lack of confidence in PPE adequacy (*χ* ^2^(2) = 74.3; *p* = .001) and intent to leave (Table 3). We also observed a significant difference between high levels of physical exhaustion and intent to leave (*χ* ^2^(2) = 57.8; *p* = .001). We also observed significant differences in the ordinal measurement of burnout when stratified by intent to leave, such that nurses who intended to leave had a greater presence of burnout compared with nurses who did not intend to leave (*χ* ^2^(2) = 507.5; *p* = .001).

**TABLE 3:** PPE inadequacy, physical exhaustion, and burnout by intent to leave

	Intent to leave	%	No intent to leave	%	*χ* ^2^
**Lacked PPE**					
No	95	8.5	229	15.0	26.0*
Yes	1017	91.5	1301	85.0	
**Rationed and reused PPE**					
No	33	3.0	75	4.9	6.1
Yes	1079	97.0	1455	95.1	
**Lack of confidence in PPE protection**					
No	229	20.6	526	34.4	61.3*
Yes	883	79.4	1004	65.6	
**Lack of confidence in PPE adequacy**					
No	131	11.8	382	25.0	74.3*
Yes	981	88.2	1148	75.9	
**Physical exhaustion**					
No	150	13.5	391	25.6	57.8*
Yes	962	86.5	1139	74.4	
**Ordinal burnout**					
I enjoy my work. I have no symptoms of burnout	25	2.3	151	9.9	507.5*
Occasionally I am under stress, and I don't always have as much energy as I once did, but I don't feel burned out	188	16.9	675	44.3	
I am definitely burning out and have one or more symptoms of burnout, such as physical and emotional exhaustion	396	35.7	517	33.9	
The symptoms of burnout that I'm experiencing won't go away. I think about frustration at work a lot	275	24.8	146	9.6	
I feel completely burned out and often wonder if I can go on. I am at the point where I may need some changes or may need to seek some sort of help	226	20.4	35	2.3	
**Dichotomized burnout**					
No	213	19.2	826	46.0	329.4*
Yes	899	80.8	704	54.0	

*Note*: *n* = 2642 for relationship between dichotomized burnout and intent to leave.

Abbreviation: PPE, personal protective equipment.

* *p* = .001.

### 4.5 Predictors of burnout

Controlling for covariates, in the multivariable regression analyses estimating the odds of burnout, we found no significant relationships between staffing and burnout (Table 4). However, RNs who reported lack of confidence in PPE protection had 1.59 (95% CI: 1.26–2.00, *p* = .001) times the odds of burnout compared with RNs who did not lack the confidence in PPE protection. Additionally, RNs who reported lack of confidence that the hospital had sufficient PPE had 1.77 (95% CI: 1.34–2.34, *p* = .001) times the odds of burnout compared with RNs who did not lack confidence that the hospital had sufficient PPE. Nurses who reported physical exhaustion of 7 or more on a 10‐point scale had 3.89 (95% CI: 3.19–4.76, *p* = .001) times the odds of burnout compared with RNs who did not report high levels of physical exhaustion.

**TABLE 4:** Multivariable regression analyses estimating the odds of burnout related to staffing, PPE confidence, and physical exhaustion (*n* = 2860)

Characteristic	ORs (95% CI)	*p*
**Peak staffing** ^a^	1.02 (0.99–1.06)	.222
**Lack of confidence in PPE protection**		
No	Ref	
Yes	1.59 (1.26–2.01)	.001
**Lack of confidence in PPE adequacy**		
No	Ref	
Yes	1.77 (1.34–2.34)	.001
**Physical exhaustion**		
No	Ref	
Yes	3.89 (3.19–4.76)	.001

Abbreviations: PPE, personal protective equipment; OR, odds ratio.

^a^ Adjusted for unit type, lack of PPE, rationed and reused PPE, pseudo‐*R* ^2^ = .0848.

## 5 DISCUSSION

This large cross‐sectional survey of over 3000 RNs indicates that inadequate staffing, inadequate PPE, and high levels of physical exhaustion that occurred during the first wave of the COVID‐19 pandemic contributed to over 65% of acute care RN workforce reporting burnout. As the COVID‐19 pandemic continues, empirically determining the burnout level as early as October 2020 when the COVID‐19 rates were lower than March 2020 in NJ provides evidence that burnout existed. To reduce the effects of mental health, managers need to continue to monitor frontline clinical nurses for signs of physical and emotional signs and symptoms of burnout.

Among all specialties, 64.3% of the RNs reported burnout with 36.5% of them reporting an intent to leave. Existing work confirms that burnout is a significant predictor of the intent to leave (Bourdeanu et al., 2020; Lee et al., 2020). The International Council of Nurses is reporting a global shortage of with intention to leave rates doubling to 20–30% (Nebehay, 2021). High levels of burnout are associated with adverse patient outcomes such as fatal medical errors, falls and urinary tract, and surgical site and infections (Cimiotti et al., 2012). Additionally high frequency of end‐of‐life care that occurs during the pandemic conflicted with the strong emotion regulation required of RNs may contributed to burnout (Ricou et al., 2020).

Medical/surgical RNs reported the highest intent to leave as compared with the ICU, ED, and other departments. There has always been a high intent to leave among medical/surgical RNs (Phillips, 2020). Medical/surgical nurses also reported the highest numbers of lacking, rationing, and lack of confidence in PPE adequacy as compared with the nurses in the ICUs and EDs. An additional stressor for clinicians during COVID‐19 was the fear of having to ration resources and facing limited supplies (Butler et al., 2020), which may explain burnout and intent to leave.

We found no association between staffing and burnout, which may be explained by consistent ratios prior to the pandemic and at the peak. However, in the ICUs, RNs reported an additional patient increasing the ratio from 1:2 (the accepted standard) to 1:3 COVID‐19 patients. Coupled with high physical exhaustion and inadequate PPE set the stage for burnout among these nurses. Additionally, RNs reported only the number of patients which does not account for patient acuity which may explain why we found no effect. Therefore, when preparing for future pandemics, although the number of patients may remain the same, the work environment, the lack of adequate resources, the physical exhaustion all needs to be considered to reduce burnout.

Only one out of five RNs felt confident that the hospital had adequate PPE during the first wave with medical/surgical RNs having the least confidence. The PPE inadequacy contributed to nurse burnout for all specialties. All RNs felt that their hospital lacked sufficient PPE, which was likely exacerbated by shortages of PPE nationwide. This finding highlights the need for managers to maintain adequate PPE by removing profit motives and the need to strengthen the capacity of local, state, and federal governments to effectively distribute stockpiles during future pandemics (Cohen & van der Meulen Rodgers, 2020).

All RNs reported high levels of burnout related to physical exhaustion with the highest reports in the ICUs. This may be due to proning of ventilated patients as well as donning and doffing PPE several times a shift. There were significant associations between the prevalence of physical symptoms and psychological outcomes among health care workers during COVID‐19 (Chew et al., 2020) and the compounding effect of suppressing emotions while working the frontlines during one of the world's toughest health crisis likely increased burnout.

### 5.1 Limitations

There are several limitations of this study. First, this survey was a snapshot of perceptions that occurred in October 2020, and due to the cross‐sectional design of the study, we cannot infer causality. Longitudinal studies measuring burnout levels are needed to examine the effect of the pandemic on nurse burnout throughout this protracted period of time. This study was conducted among licensed RNs in NJ which limits the generalizability to other geographic areas and health care settings. Additionally, the response rate may have been higher if acute care nurses were recruited directly through hospitals rather than emailing all NJ licensed RNs. However, we felt that recruiting RNs through the hospital may have created a sense of compulsion for the participants. Regarding measures, the burnout measure was psychometrically tested in primary care, but limited in acute care and we lost of granularity of the ordinal burnout variable by dichotomizing it to run our models. We also recognize that the evidence was limited at the time in presenting psychometrics for PPE adequacy; therefore, we created our own measures for PPE adequacy that was based on a subjective rating of PPE adequacy, which is a threat to the overall study's validity. Further, the overall survey was self‐administered and may be subject to self‐report bias and selection bias in that RNs that participated in the study may have been more affected by COVID‐19.

## 6 CONCLUSIONS

During the peak of the first wave of the COVID‐19 pandemic, staffing, PPE inadequacy, and physical exhaustion all contributed to frontline, acute care nurse burnout. This burnout was associated with higher levels of intent to leave the profession. Mental health interventions are vital to nurse well‐being as well as managers working with nursing professional organizations for relief funds to reduce the effects of burnout.

## 7 IMPLICATIONS FOR NURSING MANAGEMENT

Burnout is an international problem facing all nurses during the COVID‐19 pandemic. Our findings support existing evidence that demonstrate an immediate need for proactive planning for crisis management and mental health assistance to meet provider needs (Butler et al., 2020). Immediate interventions include training managers to recognize the mental health effects of their frontline clinical staff and to refer them to employee assistance programs. Other strategies include supporting nurses with wellness seminars and conversations to decrease moral distress when caring for COVID‐19 patients (Hofmeyer & Taylor, 2021). These efforts need to be sustained and supported by hospitals. Block scheduling may also allow nurses to recover from the effects of physical exhaustion.

Nurse managers should engage with nursing professional specialty organizations and work with them to identify grassroot initiatives to reduce burnout. Some nurse organizations have committed their mission to improve frontline nurse health, encourage peer‐to‐peer support, and provide resources for nurses (New Jersey Nursing Initiative, 2021). Organizations such as the American Association of Critical Care Nurses for ICU RNs address issues that are specific to their membership. Managers also should elicit help from culturally based organizations like the National Association of Hispanic Nurses or the National Black Nurses Association.

Senior‐level nurse leaders should seek out COVID‐19 relief funding as an essential resource for investing in the nursing workforce. These funds can assist in developing policy initiatives such as improved staffing ratios, improving the work environment, and ensuring adequate resources. We also encourage leaders in nursing schools to train students in caring for COVID‐19 patients and invest in resiliency training during clinical rotations. Given the empirical evidence that burnout and intent to leave levels are high, leaders are charged to advocate on behalf of their staff to mitigate the burnout and intent to leave.

## CONFLICT OF INTEREST

The authors have declared no conflict of interest.

## ETHICS STATEMENT

Our work was approved by the Rutgers, the State University of New Jersey Institutional Review Board Pro 20200001775 under minimal risk and exempt category 2 based on Title 45, Part 46.101(b) of the Code of Federal Regulations.

## Data Availability

The data that support the findings of this study are available on request from the corresponding author. The data are not publicly available due to privacy or ethical restrictions.
